# Preventive effect and mechanism of compound Danshen dripping pills on contrast-induced nephropathy after percutaneous coronary interventional

**DOI:** 10.3389/fcvm.2023.1211982

**Published:** 2023-12-01

**Authors:** Han Fu, Linrui Wang, Shuo Ying, Zhicheng Zhao, Peng Zhang

**Affiliations:** ^1^ Sir Run Run Shaw Hospital, Zhejiang University School of Medicine, Hangzhou, China; ^2^ Graduate School of Tianjin Medical University, Tianjin, China; ^3^ Sheng Jing Hospital Affiliated, China Medical University, Shenyang, China; ^4^ Department of Cardiology, Tianjin Chest Hospital, Tianjin University, Tianjin, China

**Keywords:** compound Danshen dripping pills, contrast-induced nephropathy, inflammation, oxidative stress, percutaneous coronary intervention, prevention

## Abstract

**Background:**

Contrast-induced nephropathy (CIN) is one of the most common complications after coronary stent implantation due to the extensive development of coronary catheterization technology. Compound Danshen dripping pills (CDDP) are clinically used as cardiovascular drugs, relieving systemic inflammatory response. Previous studies have observed that CDDP can decrease CIN incidence after coronary stent implantation with uncertain effectiveness.

**Methods:**

We conducted a prospective, randomized, single-center, single-blind, controlled trial. We enrolled patients 18 years and older with unstable angina pectoris and NSTEMI who underwent PCI at the Tianjin Chest Hospital between November 1, 2021, and November 31, 2022, and followed for 30 days. Patients were randomized to CDDP and hydration therapy (10 capsules three times/day; *N* = 411) or hydration only (*N* = 411). The primary outcome was the contrast nephropathy incidence, defined as an elevation in serum creatinine by more than 25% or 44 μmol/L from baseline within 48–72 h of contrast exposure. Secondary outcomes included major adverse cardiovascular events post-surgery and during follow-up.

**Results:**

After 48 h of operation, the two groups had statistical significance in Scr and BUN values (80.0 ± 12.59 vs. 84.43 ± 13.49, *P* < 0.05; 6.22 ± 1.01 vs. 6.40 ± 0.93, *P* < 0.05). The difference in Scr in 72 h between the two groups was statistically significant (76.42 ± 10.92 vs. 79.06 ± 11.58, *P* < 0.05). The CIN incidence was significantly lower in the CDDP group than in the hydration group. The CIN risk was significantly elevated in patients with LVEF <50%, contrast volume ≥160 ml, and hypertension, after 48 and 72 h of operation. The serum inflammation index levels NGAL, TNF-α, oxidative stress indexes SOD, and MDA significantly differed between the two groups. However, there was no significant difference in serum apoptosis indexes Bax, Bcl-2, and Casepase-9.

**Conclusions:**

CDDP pre-treatment could prevent contrast-induced nephropathy. Inflammatory response and oxidative stress could be significant in the CDDP mechanism.

## Introduction

Advancements in percutaneous coronary intervention (PCI)-related technology have led to increased procedures annually and the enhanced use of contrast media (CM) (1). CIN is a severe complication of invasive cardiovascular procedures performed using CM (2). During angiographic or other medical procedures, CIN is a reversible acute renal failure observed after administering iodinated CM. It is the increase in serum creatinine (Scr) levels of ≥25% or an absolute increase of ≥0.5 mg/dl (44.2 mmol/L) from baseline value after 48–72 h of CM exposure (3, 4). The exact mechanism involved in CIN is unclear. However, it could be associated with hemodynamic effects, renal tubular cytotoxicity, renal medullary ischemia, hypoxia, and the inflammatory response and oxidative stress injury mediated thereby (5–8). CIN incidence in patients with normal renal function is not high. In contrast, factors such as advanced age, diabetes, and high-dose CM could elevate CIN incidence.

CDDP is a pure Traditional Chinese medicine developed using modern high-tech means, which has been used for 20 years to treat coronary heart disease (9). Its main components include Salvia miltiorrhiza, Panax notoginseng, and Dipterocarpaceae (10). Modern pharmacological studies revealed that CDDP has antioxidant and anti-inflammatory properties. They also protect endothelial function, inhibit platelet adhesion, increase coronary vascularity, and improve microcirculation (11). Recently, CDDP has been frequently used to treat CHD sufferers combined with PCI. Previous studies have established that CDDP pre-treatment can decrease CIN occurrence in PCI patients, indicating that early use of CDDP before PCI is appropriate adjuvant drug therapy. However, the mechanism of action on CDDP to prevent CIN remains unclear (12). CDDP mechanism was further analyzed to avoid CIN by detecting inflammatory indicators of apoptosis and oxidative stress indicators before and after PCI.

## Materials and methods

### Study design

This is a prospective, randomized, single-center, single-blinded controlled trial with a parallel 2-arm group undertaken in the Department of Cardiology, Tianjin Chest Hospital, to determine the feasibility and mechanism of CDDP in preventing CIN. The current study was approved by the ethics committee of Tianjin Chest Hospital and Tianjin Municipal Health Commission (number: 2021205). All the patients or their next of kin provided written informed consent before enrolment or the subsequent registry phase. The independent trial steering, data monitoring, and ethics committees provided trial oversight. Our study followed the Consolidated Standards of Reporting Trials reporting guidelines.

### Study population

The study enrolled patients who aged 18 years or older with unstable angina pectoris and acute non-ST-segment elevation myocardial infarction (NSTEMI) treated with intravascular iodine contrast for PCI at the Third Department of Cardiology, Tianjin Chest Hospital from November 1, 2021 to November 31, 2022. The last participant completed a 30-day follow-up in December 2022. Exclusion patients included individuals with acute ST-segment elevation myocardial infarction (STEMI) receiving emergency PCI, uncontrolled hypertension (treated systolic blood pressure >160 mm Hg or diastolic blood pressure >100 mm Hg), receiving CM 14 days before PCI, using any renal toxicity drugs during the perioperative period, severe renal and cardiac insufficiency (GFR <30 ml/min or creatinine clearance <30 ml/min or LVEF <30%), cardiogenic shock and heart failure, hypersensitivity to CM, severe liver damage, autoimmune diseases, malignant tumor, fever, participation in another randomised trial or isolation (close contact with the COVID-19 patient).

### Sample size

Based on previous trial data, we projected the necessary sample size indicating that 13.38%–15% of the control and 7.7% of the CDDP groups would develop CIN (12, 13). Assuming 10% missing primary outcome data due to death or missed assessments, the total sample size of 706 participants would have a statistically significant difference, with 90% power and a two-sided *α* of 0.05.

## Study protocol

Based on admission electrocardiogram (ECG) and preliminary laboratory results, 989 patients were identified as study candidates. Eligible patients who agreed to enter the study were sequentially assigned to two groups (1:1) based on computer-generated random numbers. All patients received loading doses of aspirin and clopidogrel (300 mg each) before CAG/PCI. Clinicians can decide whether to use the following medicines based on clinical requirements or guidelines, including β-antagonist, angiotensin-converting enzyme inhibitors (ACEIs)/angiotensin II receptor blockers (ARBs), calcium channel blockers (CCBs), diuretics, and a statin. Patients who underwent only CAG were excluded (mild coronary artery stenosis or severe coronary artery lesions require CABG). Patients were given the same instructions for procedure preparation and post-procedure recovery.

Patients allocated to the hydration group received only received hydration therapy (0.9% sodium chloride), because related studies have proved that hydration in the perioperative period is effective in preventing the occurrence of CIN (14). Prophylactic hydration protocols used were according to current guidelines: intravenous 0·9% NaCl 1 ml/kg per h during 12 h before and 12 h after contrast administration (at least 1,000 ml). Hydration rate was reduced to 0.5 ml/kg/h for patients with LVEF ≤45%. Patients in the CDDP group took compound Danshen Dripping Pills (Tianjin Tasly Pharmaceutical Group Co., Ltd., specification: 27 mg/capsule) orally 2 days before surgery at the same time as the hydration therapy, 10 capsules/time, 3 times/day; continuous oral administration after surgery, 10 capsules/time, 3 times/day, for 14 day. Iodoprolamide (Bayer medical and health care Co., Ltd, Leverkusen, Germany) contrast agent was used in all patients during PCI.

### Data collection and management

An independent physician and a data safety monitoring board periodically assessed safety throughout the study. The attending physician was responsible for the clinical management of the patient. Blood samples of patients were obtained at admission, 48 h, and 72 h after CM to determine the Scr or BUN level. Part of the blood sample was centrifuged, and the serum was taken and stored in a −20°C refrigerator. Serum creatinine was measured in umol/L, and the serum Scr content of all samples was determined using the same automatic large-scale biochemical test analyzer (Japan TOSHIBA).

The laboratory staff handling the serum samples were blinded to the treatment or patient identity. The samples were affixed with coded tags. Doctors who carried out the CAG/PCI remained undercover for treatment assignment. All the basic patient information, blood biochemical examination, and PCI data were obtained using a special database.

### Serum enzyme-linked immuno sorbent assay detection

Enzyme-linked immunosorbent assay (ELISA) was used to detect the contents of inflammatory factors (NGAL, TNF-α), apoptosis factors (Bcl-2, Bax, Caspase-9) and oxidative stress factors (SOD, MDA). The kit was provided by the elixir pharmaceutical company of Canada (Elixir Canada Medicine Company Ltd.).

The ELISA procedure was as follows: The EP tube was marked and placed symmetrically in the centrifuge after balancing, centrifuged at 4°C at 3,000 RPM for 10 min, then the supernatant was absorbed into the new tube and the precipitation was discarded. Put the supernatant into the −80°C freezer for later use. Dispensed 100 μl of standards, specimens and controls into appropriate wells, dispensed 50 μl of Enzyme Conjugate into each well, incubated at thermostat 37°C for 60 min; rinsed and flicked the micro wells three times with 300 μl diluted wash concentrate; dispensed 50 μl of Chromogen A and Chromogen B reagent into each well, mixed for 5 s, incubated at 37°C in the dark for 20 min; stopped the reaction by adding 50 μl of stop solution to each well, read absorbance at 450 nm with a micro plate reader within 10 min (using multiskan MK3 system, Thermo Electron Corporation, Massachusetts, United States).

### Study endpoints

The primary outcome measure was CIN, which was determined based on elevated Scr levels after exposure to CM for 48 and 72 h. The highest Scr on postcontrast hours 48 or 72 was used to calculate the change in serum creatinine (the primary end point). The other clinical endpoints included MACE such as all-cause mortality, unstable angina pectoris, acute heart failure, malignant arrhythmia (ventricular fibrillation, ventricular tachycardia, and polymorphic ventricular premature), acute myocardial infarction, and cerebrovascular events, during hospitalization and after 1 month follow-up.

### Statistical analysis

Results are expressed as numbers (%) or mean ± SD. Categorical data were presented as frequencies and percentages and analyzed using 2-tailed *χ*^2^ tests, and continuous variables were compared using an unpaired *t*-test. Fisher's exact test was used to compare percentages when the expected frequency was <5. Univariable and multivariable logistic regression analysis was used to exclude the influence of confounding factors, and high-risk confounders were selected to calculate the relative risk (RR) of 95% CIs using the log-binomial model for the categorical variables. A Kaplan–Meier survival curve was used to analyze the relationship between CDDP and postoperative MACE events. All statistical tests were 2-sided and *P* < 0.05 was considered to be statistically significant. No imputation techniques were applied for missing data. Statistical analyses were performed using R (R software 4.2.2).

## Results

**Figure 1:**
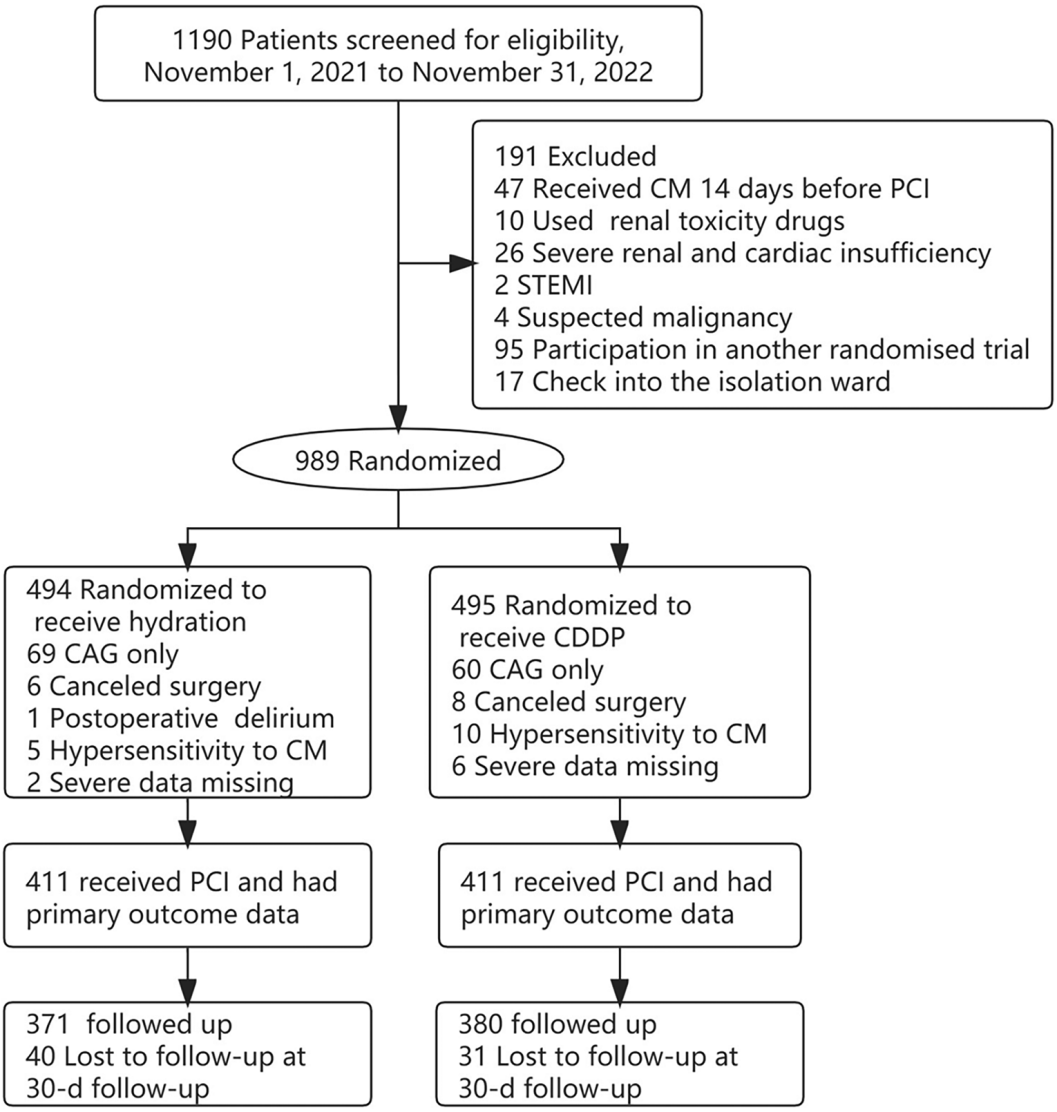
Patient flowchart. CM, contrast media; CAG, coronary arteriography; PCI, percutaneous coronary intervention; STEMI, st elevated myocardial infarction; CIN, contrast-induced nephropathy.

Of the 1,190 patients initially enrolled in the study, 47 were treated with CM 14 days before PCI, 10 were treated with nephrotoxic drugs, 26 had severe renal and cardiac dysfunction, 2 had STEMI after admission, 4 had suspected malignancies, 95 were enrolled in another randomized trial, and 17 were admitted to an isolation unit. 989 patients were randomly divided into hydration group and CDDP group. CAG test alone was excluded, and patients with postoperative delirium, CM allergy and severe data loss were excluded. A total of 822 patients were included for statistical analysis and 751 were followed up (Figure 1). A total of 29 cases of MACE occurred in both groups during 30 days of follow-up (Hydration group: 15 cases of unstable angina pectoris, 1 case of acute heart failure; CDDP group: 1 case of cerebrovascular event, 12 cases of unstable angina pectoris) The incidence of MACE in 751 follow-up patients showed no significant difference between the two groups.

### Baseline clinical characteristics

There were no significant differences in the baseline characteristics between the two groups (age, sex, BMI, hypertension, diabetes, cerebrovascular disease, left ventricular ejection fraction LVEF, NSTEMI, contrast volume, hydration amount, triglyceride, total cholesterol, high-density lipoprotein cholesterol, and low-density lipoprotein cholesterol levels, the use of ACEI/ARB, diuretic, beta-blockers and CCBs) before operation (Table 1).

**Table 1:** Comparisons of baseline characteristics between the two groups.

Variables	Hydration group	CDDP group	*P*
Age (years)	66.69 ± 7.86	66.40 ± 7.10	0.597
Male (%)	200 (48.7)	207 (50.4)	0.676
BMI (kg/m^2^)	24.70 ± 2.22	24.89 ± 2.00	0.191
Hypertension (%)	309 (75.3)	306 (74.5)	0.872
Diabetes (%)	92 (22.4)	104 (25.3)	0.370
Cerebrovascular disease (%)	61 (14.8)	50 (12.2)	0.368
NSTEMI (%)	61 (14.8)	68 (16.5)	0.565
LVEF (%)	59.82 ± 6.86	59.58 ± 6.91	0.616
Contrast volume (ml)	163.80 ± 50.43	165.18 ± 55.23	0.707
Hydration amount (ml)	1,273.67 ± 272.58	1,251.81 ± 283.40	0.260
TC (mmol/L)	4.37 ± 0.69	4.43 ± 0.68	0.146
TG (mmol/L)	1.70 ± 0.87	1.76 ± 0.98	0.387
LDL-C (mmol/L)	2.42 ± 0.69	2.41 ± 0.59	0.823
HDL-C (mmol/L)	1.24 ± 0.33	1.25 ± 0.34	0.413
ALT (U/L)	25.63 ± 11.59	26.94 ± 11.40	0.100
AST (U/L)	23.26 ± 9.58	22.52 ± 5.64	0.175
Homocysteine (umol/L)	11.34 ± 4.26	11.55 ± 5.54	0.560
Hypokalemia (mmol/L)	4.11 ± 0.37	4.07 ± 0.35	0.550
ACEI/ARB (%)	300 (73.0)	280 (68.1)	0.146
diuretic (%)	63 (15.3)	77 (18.7)	0.228
β-antagonist (%)	321 (78.1)	315 (76.6)	0.677
CCB (%)	88 (21.4)	107 (26.0)	0.140

Data are expressed as mean ± SD or *n* (%). BMI, body mass index; NSTEMI, non-st elevation myocardial infarction; LVEF, left ventricular ejection fraction; TC, total cholesterol; TG, triglycerides; HDL-C, high-density lipoprotein cholesterol; LDL-C, low-density lipoprotein cholesterol; ACEI, angiotensin-converting enzyme inhibitor; ARB, angiotensin receptor blocker; CCB, calcium channel blockers; ALT, alanine transaminase; AST, aspartate transaminase.

### Comparison of Scr, BUN levels and CIN incidence in the two groups

**Figure 2:**
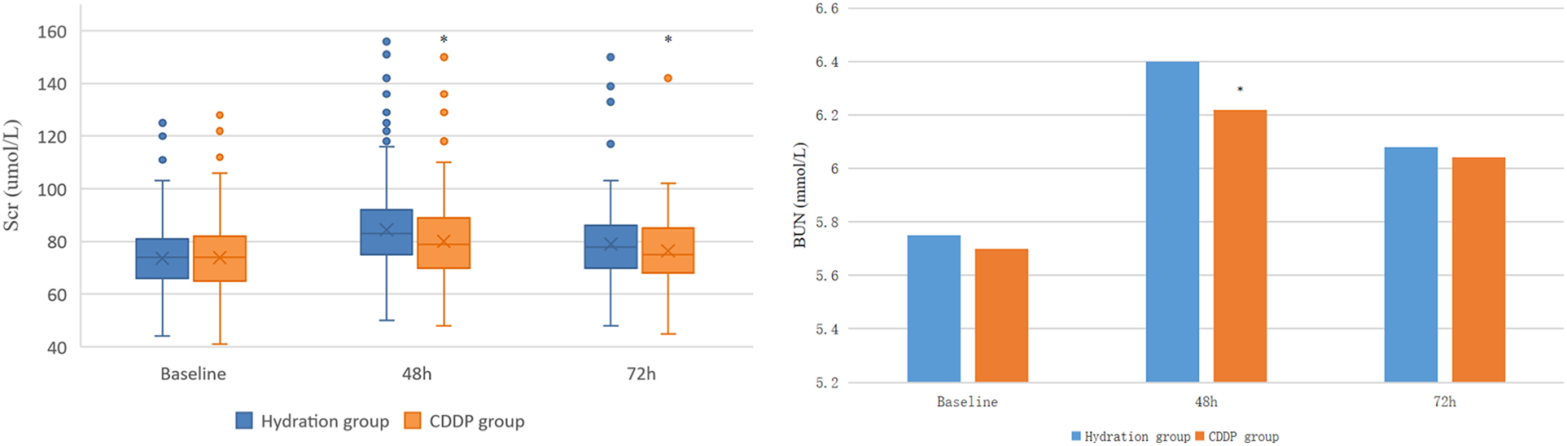
Comparison of Scr and BUN in two groups. Scr, Serum creatinine; BUN, blood urea nitrogen. Values are expressed as mean ± SD. The points in the box plot are data outliers, which account for a small percentage of the total, and there is no obvious difference between the statistical results after excluding the outliers and the calculation and analysis process when the outliers are included in the data. *Compared with the hydration group, *P* < 0.05.

The two groups had no significant differences in baseline Scr and BUN values. Scr and BUN values were significantly higher than the baseline in the two groups after 48 and 72 h of operation. After 48 h and 72 h of contrast agent injection, the Scr value of the CDDP group was significantly lower than the hydration group (80.0 ± 12.59 vs. 84.43 ± 13.49, *P* < 0.05; 76.42 ± 10.92 vs. 79.06 ± 11.58, *P* < 0.05, Figure 2A). Similarly, the BUN value of the 48 h CDDP group was lower than the hydration group, with a statistically significant difference (6.22 ± 1.01 vs. 6.40 ± 0.93, *P* < 0.05, Figure 2B).

**Figure 3:**
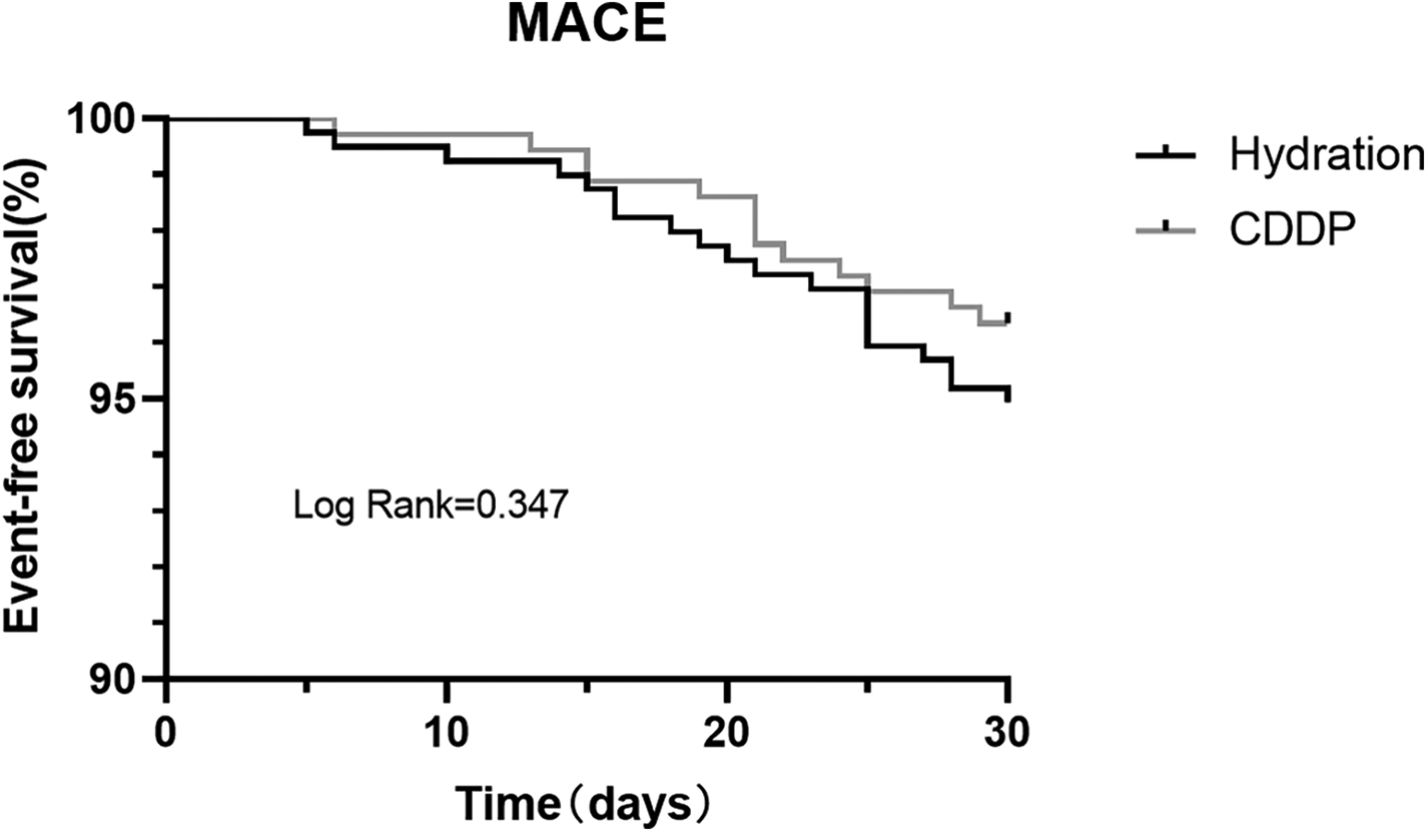
Kaplan–Meier survival curve for MACE in two groups. MACE, major adverse cardiovascular events.

CIN incidence in the two groups was 11.2% and 6.6%, respectively (*P* = 0.027). Univariable logistic regression analysis was used to analyze the factors affecting CIN. CIN was chosen as the dependent variable, and factors that could affect CIN development (Age, Male, Hypertension, Diabetes mellitus, Myocardial infarction, Cranial vascular disease, LVEF, Contrast volume, Hydration amount, BMI, LDL, Hypokalemia, ACEI/ARB, Diuretic, β-antagonist, CCB and CDDP) were considered as independent variables. Multivariate logistic regression analysis was performed on the factors that had significant influence on CIN in the univariate analysis [OR: 0.539; 95% CI: 0.325–0.892, *P* = 0.016)] (Table 2). The log-binomial model estimated the association between patient factors, CDDP, and hydration treatments. Patients taking CDDP were less likely to develop CIN in adjusted models than those treated with hydration alone (RR: 0.58; 95% CI: 0.37–0.91). Patients with LVEF <50% were more likely to develop CIN than patients with LVEF ≥50% (RR: 2.38; 95% CI: 1.31–4.29). Moreover, 160 ml contrast volume was more likely to develop CIN (RR: 1.74; 95% CI: 1.09–2.79). Similarly, hypertensive patients were more likely to develop CIN (RR: 1.88; 95% CI: 1.02–3.49). No association could be obtained between age or sex and CIN incidence (Table 3). Kaplan–Meier survival analysis revealed no significant difference in the cumulative incidence of MACE between the two groups. There was a change trend between hydration group and CDDP group in event-free survival; the event-free survival of hydration group was lower than that of CDDP group (Log-rank test, *P* = 0.347, Figure 3).

**Table 2:** Univariable and multivariable logistic regression analysis for certain confounding factors of CIN.

Variables	Univariable analysis		Multivariable analysis	
	OR (95% CI)	*P*	OR (95% CI)	*P*
Age (years)	0.986 (0.956–1.016)	0.345		
Male	0.778 (0.480–1.263)	0.310		
Hypertension	1.998 (1.031–3.871)	0.040*	2.044 (1.046–3.994)	0.036*
Diabetes mellitus	1.643 (0.980–2.756)	0.060	1.436 (0.838–2.465)	0.188
Myocardial infarction	0.737 (0.357–1.521)	0.409		
LVEF (mmol/L)	0.959 (0.929–0.990)	0.010*	0.958 (0.927–0.989)	0.009*
Cranial vascular disease	1.292 (0.672–2.484)	0.443		
Contrast volume (ml)	1.006 (1.002–1.010)	0.003*	1.006 (1.002–1.010)	0.007*
Hydration amount (ml)	1.000 (0.999–1.001)	0.441		
BMI (kg/m^2^)	1.011 (0.902–1.133)	0.853		
LDL (mmol/L)	0.749 (0.514–1.091)	0.133		
Hypokalemia (mmol/L)	0.813 (0.415–1.594)	0.547		
ACEI/ARB	0.767 (0.440–1.336)	0.349		
Diuretic	0.942 (0.502–1.768)	0.853		
β-antagonist	1.132 (0.647–1.980)	0.664		
CCB	0.696 (0.411–1.181)	0.179		
CDDP	0.558 (0.340–0.916)	0.021*	0.539 (0.325–0.892)	0.016*

LVEF, left ventricular ejection fraction; BMI, body mass index; LDL-C, low-density lipoprotein cholesterol; ACEI, angiotensin-converting enzyme inhibitor; ARB, angiotensin receptor blocker; CCB, calcium channel blockers.

* *P* < 0.05, the difference is statistically significant.

**Table 3:** Characteristics associated with CIN.

Characteristic	Hydration group (*n* = 411)	CDDP group (*n* = 411)	Log binominal Analysis	
			RR (95% CI)	*P*
CDDP	–	–	0.58 (0.37–0.91)	0.018*
Age (years), No. (%)				
<65	155 (37.7)	160 (38.9)	1 [Reference]	
≥65	256 (62.3)	251 (61.1)	0.87 (0.54–1.28)	0.403
Sex, No. (%)				
Female	211 (51.3)	206 (50.1)	1 [Reference]	
Male	200 (48.7)	205 (49.9)	0.81 (0.54–1.29)	0.421
LVEF, No. (%)				
≥50%	388 (94.4)	377 (91.7)	1 [Reference]	
<50%	23 (5.6)	33 (8.0)	2.38 (1.31–4.29)	0.004*
Contrast volume (ml), No. (%)				
<160	177 (43.1)	188 (45.7)	1 [Reference]	
≥160	234 (56.9)	223 (54.3)	1.74 (1.09–2.79)	0.020*
Hypertension, No. (%)	309 (75.2)	306 (74.5)	1.88 (1.02–3.49)	0.040*

LVEF, left ventricular ejection fraction.

* *P* < 0.05, the difference is statistically significant.

### Determination of apoptotic, inflammatory and oxidative stress indicators

**Figure 4:**
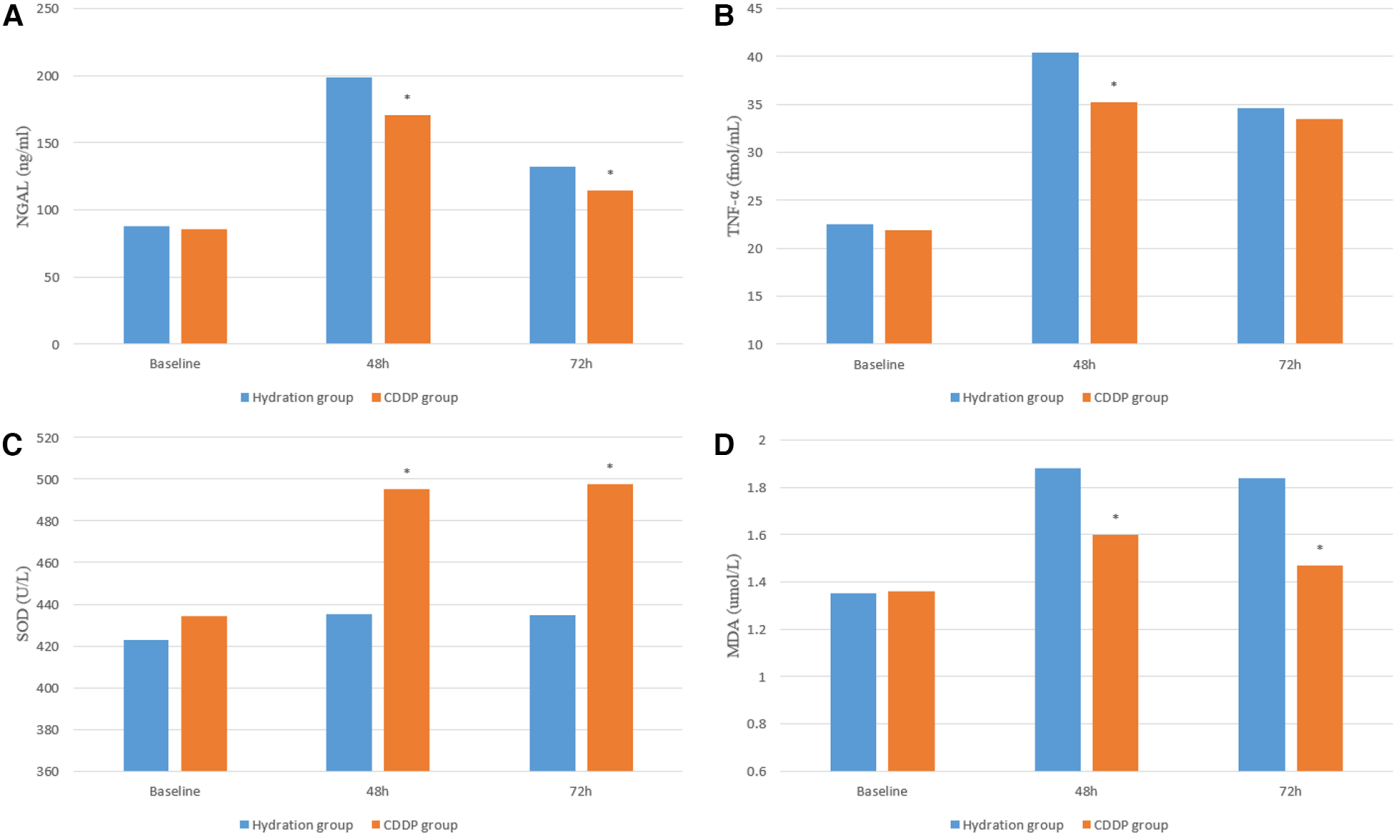
Serum inflammation and oxidative stress were detected by ELISA. NGAL, neutrophil gelatinase-associated lipoprotein; TNF-α, tumor necrosis; SOD, Super oxygen dehydrogenises; MDA, Malondialdehyde. Values are expressed as mean ± SD. *Compared with the hydration group, *P* < 0.05.

**Figure 5:**
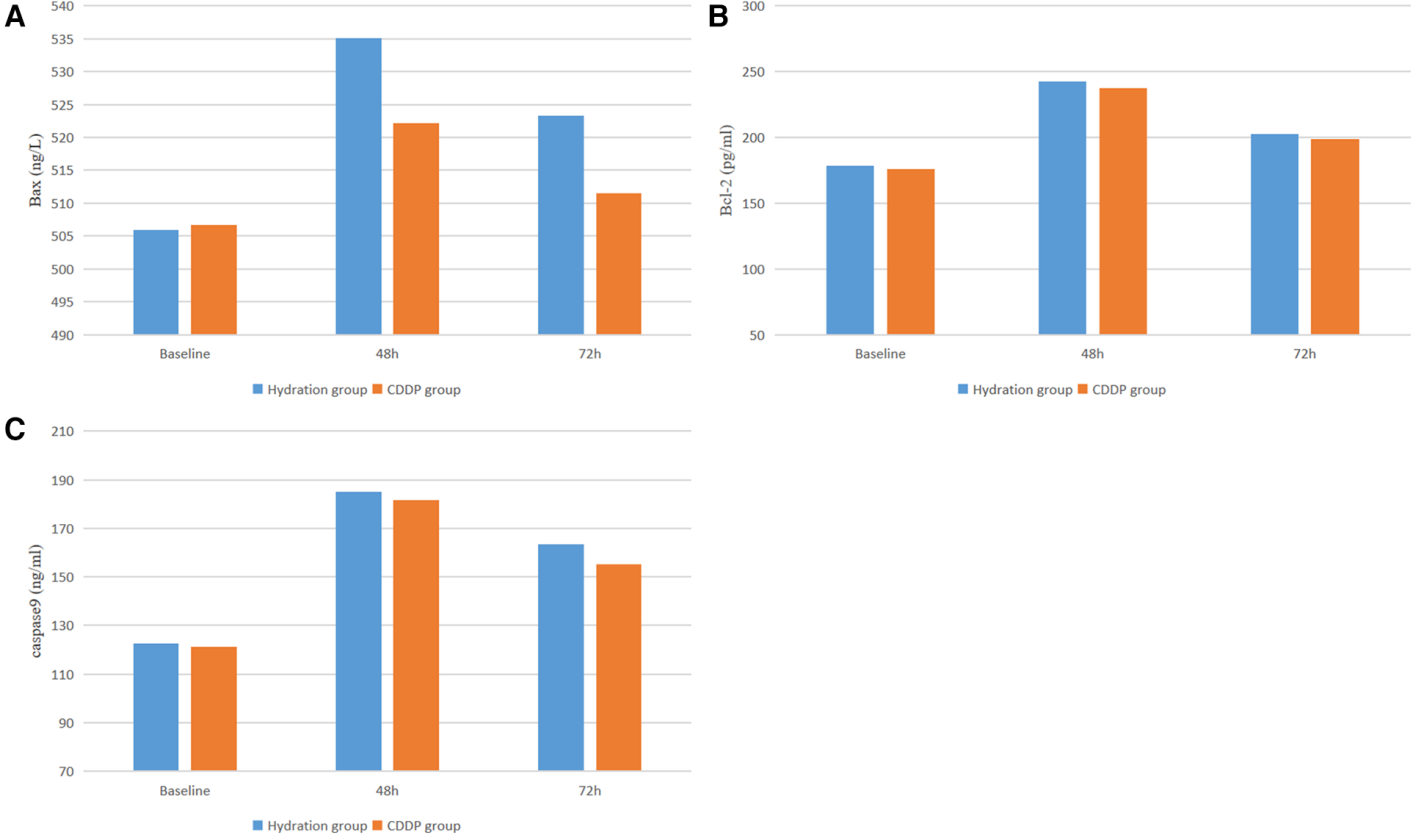
Serum apoptosis were detected by ELISA. BAX, Bcl-as-sociated X protein; Bcl-2, B-cell lymphoma-2. Values are expressed as mean ± SD. *Compared with the hydration group, *P* < 0.05.

After 48 and 72 h of surgery, the levels of NGAL and kidney injury markers increased in both groups. NGAL levels were significantly lower in the CDDP group than those in the hydration group after 48 h of surgery (132.15 ± 58.46 vs. 198.54 ± 89.21, *P* < 0.05; 114.47 ± 43.75 vs. 170.47 ± 71.17, *P* < 0.05, Figure 4A). Moreover, the level of inflammatory mediators TNF-α in the CDDP group was significantly lower than in the hydration group (35.23 ± 14.56 vs. 40.38 ± 14.37, *P* < 0.05, Figure 4B). Thus, inflammatory damage occurred at 48 h and 72 h after contrast nephropathy, causing mild renal degeneration. At 48 h and 72 h after injection of CM, the oxidative stress index SOD level in the CDDP group was higher than that in the hydration group (435.02 ± 101.74 vs. 495.11 ± 115.95, *P* < 0.05; 434.95 ± 121.10 vs. 497.46 ± 115.77, *P* < 0.05, Figure 4C), while the MDA level was significantly lower in the CDDP group than in the hydration group (1.35 ± 0.35 vs. 1.88 ± 0.54, *P* < 0.05; 1.36 ± 0.38 vs. 1.60 ± 0.48, *P* < 0.05, Figure 4D). Bax, Bcl-2, and Caspase-9 were lower than those in the hydration group at 48 h and 72 h, with no statistical significance (*P* > 0.05, Figure 5), indicating that inflammation and oxidative stress were essential CIN occurrence mechanisms.

## Discussion

This study evaluated the association between perioperative CDDP use and CIN incidence in patients with non-ST-segment elevation acute coronary syndromes demanding interventional treatment with contrast agents. This prospective, randomized, controlled trial revealed that CDDP combined with hydration therapy significantly decreased the risk of CIN through anti-inflammatory and antioxidant stress effects. Moreover, LVEF < 50%, CM ≥ 160 ml, and hypertension are CIN high-risk factors in patients.

Procedures with intravascular iodinated contrast material pose a risk to renal function, especially in patients with compromised renal functions. CIN is a frequent complication after intravascular CM administration. It is the third most common cause of acute kidney injury among hospitalized patients, second only to ischemic and drug-induced injuries (4). Patients with CIN have a greater risk for non-renal complications, such as cardiac, vascular, and systemic problems. Treatment is limited to supportive measures in patients who develop CIN until renal impairment resolves (15). Many studies have shown that inflammation and oxidative stress are necessary for renal dysfunction and deterioration. CIN can be regarded as renal impairment due to CM, which stimulates the response of pro-inflammatory factors, including tumor necrosis factor, interleukin-6, and IL-1 family cytokines *in vivo*. This directly leads to renal tubular epithelial cell damage and an elevated risk of CIN (16, 17). CM with high viscosity characteristics can lead to toxicity to renal tubule cells. Moreover, CM affects the blood oxygen delivery by renal tubule cells, triggers the release of reactive oxygen species, and elevates oxidative stress and free radical formation. This damages the cell membrane and nucleic acid and consumes NO, leading to enhanced oxygen consumption (18).

CDDP is a pure Chinese medicine drop with high efficiency, quick effect, and multi-effect developed by modern pharmaceutical technology. The main chemical components have three traditional Chinese medicines (19): Salvia miltiorrhiza, pseudo-ginseng, and borneol. Salvia miltiorrhiza has water-soluble tanshinol, salvianolic acid B, and protocatechualdehyde as active ingredients. Water-soluble tanshinol can reduce platelet aggregation and possesses various pharmacological effects. These include anticoagulation, lipid regulation, calcium ion antagonism, fibroblast proliferation and matrix secretion inhibition, and anti-inflammatory effects by inhibiting cell surface adhesion molecules. Total saponins are the active ingredients of Panax notoginseng extracted from Panax notoginseng. Borneol is made of borneol gum with main functions such as heat-clearing, deswelling, and detumescence. Modern pharmacological studies indicate that borneol has anti-inflammatory and antibacterial effects (20–23). Moreover, the potential mechanism of CDDP on myocardial ischemia involves energy metabolism regulation. This includes regulating the primary energy production mode of ischemic heart tissue, thereby inducing metabolic transformation to fatty acid metabolism (24).

ACS patients suffer from excessive inflammation and endothelial dysfunction, ongoing platelet aggregation, cell adhesion, and microcirculation obstruction after PCI. NGAL and TNFα are highly sensitive inflammatory markers, indicating the inflammatory state of the body. NGAL is a relatively small protein of the lipocalcin family that is the response of the nephron to tubular epithelial damage (25). Recent evidence indicates that NGAL levels are enhanced and stable in acute kidney injury, readily detectable in serum (26, 27). Thus, elevated NGAL levels could predict CKD progression and serve as an early kidney damage marker (28). NFα is mainly composed of monocytes and macrophages. TNF-α inhibits the expression of endothelial nitric oxide synthase (eNOS) in endothelial and vascular smooth muscle cells. This increases the NF-κB pathway activation through eNOS mRNA instability and NADPH oxidase activation to induce inflammation (29–31). Reactive oxygen species are products of aerobic metabolism and are involved in different pathological states of acute kidney injury (AKI) along with mitochondrial oxidative stress and autophagy. Enzyme and non-enzyme defense systems in mitochondria eliminate excess ROS to protect cells from oxidative stress (32). SOD and others constitute the scour enzyme system regulating mitochondrial ROS. Thus, SOD converts superoxide anions (the most dangerous ROS produced) into hydrogen peroxide and eventually into H_2_O (33, 34). Therefore, the serum levels of NGAL and TNF*α* significantly differed between the two groups at 48 h after applying CM. NGAL levels were higher in the hydration group than those in the CDDP group at 48 h and 72 h, indicating different degrees of inflammatory damage to kidney function. SOD and MDA contents were higher in the hydration group than those in the CDDP group at 48 h and 72 h, depicting that oxidative stress was involved in affecting renal function. Bax, Bcl-2, and Caspase-9 decreased at 48 h and 72 h in the CDDP group with a changing trend but no statistical significance (*P* > 0.05).

At present, many animal experiments have been conducted to explore the pathogenesis of CIN. Some studies have found that NGAL levels in CIN rat models exposed to dehydration and CM are significantly increased, and the renal tubule ultrastructure changes are serious, which are represented by mitochondrial swelling, mitochondrial membrane rupture, and ridge disappearance (35). By reducing rat food intake by 40% 4 weeks prior to CM injection, it was found that caloric restriction (CR) reduced CM-induced apoptosis, ROS, and inflammation in rat extrinsic medulli via the SIRT1/GPX4 pathway (36). In addition, the degree of oxidative stress was different in different anatomical renal areas. Evaluation of blood flow and histopathology in different anatomical renal areas (medullary, cortical, and perimedullary) in New Zealand rabbits showed that renal toxicity was clearly confined to the cortex and extramedullary, with characteristic tubular necrosis and vacuolation. The medullary areas appear to be more widely but less severely affected, and the REDOX status of the cortical areas is severely affected (37). Cytological observation of touch preparation technique (TPT) also showed that CM-induced ROS production, apoptosis and cell degeneration led to renal cytopathosis (38). Studies have found that the kidney protection effect of some commonly used clinical drugs is mainly through the antioxidant effect to prevent kidney toxicity (39, 40). These results are similar to those of our previous animal studies (7, 8).

The risk factors for coronary heart disease still exist after PCI. When the infarct vessel recanalization, it will also cause certain damage to the vascular endothelium, resulting in the recurrence of angina pectoris, arrhythmia, heart failure and other major vascular adverse events, which will bring adverse effects on the prognosis of patients (41). CDDP can inhibit platelet aggregation, reduce inflammation and damage of vascular endothelium. At present, a number of studies have evaluated the reduction of CDDP in the incidence of cardiovascular adverse events after PCI (42). Rong Y et al. observed that CDDP preconditioning could decrease the incidence of CIN in patients with PCI. Thus, CDDP is an appropriate adjuvant drug therapy before PCI (1). Zhang Y et al. also confirmed that CDDP could decrease CIN incidence in patients with PCI and the incidence of major cardiovascular adverse events (43). These were similar to our findings. Simultaneously, the mechanism of reducing the incidence of CIN by CDDP was further discussed, which could be connected with reducing inflammation and oxidative stress.

Our study has some limitations. First, we excluded patients with severe renal insufficiency and those who received CM 14 days before the procedure, as well as patients with severe cardiac insufficiency, heart failure, malignant tumors, fever, and emergency PCI. Second, the study was a single center with participants from the same hospital.

## Data Availability

The raw data supporting the conclusions of this article will be made available by the authors, without undue reservation.

## References

[1] Bhatt DL. Percutaneous coronary intervention in 2018. JAMA. (2018) 319(20):2127–8. 10.1001/jama.2018.5281 29800163

[2] Cuddy E Robertson S Cross S Isles C. Risks of coronary angiography. Lancet. (2005) 366(9499):1825. 10.1016/S0140-6736(05)67729-X 16298222

[3] Mamoulakis C Tsarouhas K Fragkiadoulaki I Heretis I Wilks MF Spandidos DA Contrast-induced nephropathy: basic concepts, pathophysiological implications and prevention strategies. Pharmacol Ther. (2017) 180:99–112. 10.1016/j.pharmthera.2017.06.009 28642116

[4] Mehran R Nikolsky E. Contrast-induced nephropathy: definition, epidemiology, and patients at risk. Kidney Int. (2006) 69:S11–5. 10.1038/sj.ki.5000368 16612394

[5] Kusirisin P Chattipakorn SC Chattipakorn N. Contrast-induced nephropathy and oxidative stress: mechanistic insights for better interventional approaches. J Transl Med. (2020) 18(1):400. 10.1186/s12967-020-02574-8 33081797 PMC7576747

[6] Geenen RWF Kingma HJ van der Molen AJ. Contrast-induced nephropathy: pharmacology, pathophysiology and prevention. Insights Imaging. (2013) 4(6):811–20. 10.1007/s13244-013-0291-3 24092564 PMC3846935

[7] Zhang H Zhang P Zhang X Song Y Zeng Z Fu X Novel nanoliposomes alleviate contrast-induced acute kidney injury in New Zealand rabbits by mediating inflammatory response. Ann Transl Med. (2021) 9(15):1250. 10.21037/atm-21-3201 34532387 PMC8421945

[8] Zhang P Zhang X Zhang J Song Y Liu T Zeng Z Novel nanoliposomes alleviate contrast-induced nephropathy by mediating apoptosis response in New Zealand rabbits. Front Mol Biosci. (2021) 8:681849. 10.3389/fmolb.2021.681849 34295921 PMC8290201

[9] Lv C Liu C Liu J Li Z Du X Li Y The effect of compound Danshen dripping pills on the dose and concentration of warfarin in patients with various genetic polymorphisms. Clin Ther. (2019) 41(6):1097–109. 10.1016/j.clinthera.2019.04.006 31053296

[10] Zou HM Zhang B Xu XC Su J Sun YN Xue S Urinary metabolomic strategy to evaluate compound Danshen dripping pills for myocardial ischaemia in rats. J Pharm Biomed Anal. (2015) 112:98–105. 10.1016/j.jpba.2015.04.033 25974727

[11] Zhou W Yuan WF Chen C Wang SM Liang SW. Study on material base and action mechanism of compound Danshen dripping pills for treatment of atherosclerosis based on modularity analysis. J Ethnopharmacol. (2016) 193:36–44. 10.1016/j.jep.2016.07.014 27396350

[12] Yang R Chang L Guo B Wang Y Wang Y Jin X Compound Danshen dripping pill pretreatment to prevent contrast-induced nephropathy in patients with acute coronary syndrome undergoing percutaneous coronary intervention. Evid Based Complement Alternat Med. (2014) 2014:1–6. 10.1155/2014/256268 PMC4216665 25386219

[13] Yin WJ Yi YH Guan XF Zhou LY Wang JL Li DY Preprocedural prediction model for contrast-induced nephropathy patients. J Am Heart Assoc. (2017) 6(2):e004498. 10.1161/JAHA.116.00449 28159819 PMC5523753

[14] Nijssen EC Rennenberg RJ Nelemans PJ Essers BA Janssen MM Vermeeren MA Prophylactic hydration to protect renal function from intravascular iodinated contrast material in patients at high risk of contrast-induced nephropathy (AMACING): a prospective, randomised, phase 3, controlled, open-label, non-inferiority trial. Lancet. (2017) 389(10076):1312–22. 10.1016/S0140-6736(17)30057-0 28233565

[15] Finn WF. The clinical and renal consequences of contrast-induced nephropathy. Nephrol Dial Transpl. (2006) 21(suppl_1):i2–10. 10.1093/ndt/gfl213 16723349

[16] Jaber BL Pereira BJ Bonventre JV Balakrishnan VS. Polymorphism of host response genes: implications in the pathogenesis and treatment of acute renal failure. Kidney Int. (2005) 67(1):14–33. 10.1111/j.1523-1755.2005.00051.x 15610224

[17] Bonventre JV Weinberg JM. Recent advances in the pathophysiology of ischemic acute renal failure. J Am Soc Nephrol. (2003) 14(8):2199–210. 10.1097/01.ASN.0000079785.13922.F6 12874476

[18] Goldenberg I Matetzky S. Nephropathy induced by contrast media: pathogenesis, risk factors and preventive strategies. CMAJ. (2005) 172(11):1461–71. 10.1503/cmaj.1040847 15911862 PMC557983

[19] Li Q. Research progress and clinical application of compound Danshen dripping pills. J Tradit Chin Med. (2018) 33(7):2989–91.

[20] Huang X Kou G Wang B. Progress of clinical research on compound Danshen dripping pills. Shi Zhen Chin Med. (2016) 27(5):1187–90. 10.3969/j.issn.1008-0805.2016.05.066

[21] Yang R Yao D Wang Y Chang L Li Y. Effect of compound Danshen dripping pills on cystatin C and homocysteine in patients with percutaneous coronary intervention. Henan Tradit Chin Med. (2015) 35(6):1242–5.

[22] Liu J Chen X. Effects of compound Danshen dripping pills on blood hs-CRP, P-selectin and TGF-β1 in patients with diabetic nephropathy. Chin Med Innov. (2020) 17(07):127–31. 10.3969/j.issn.1674-4985.2020.07.032

[23] Wu N Xu L. Effect of compound Danshen dripping pills combined with statins on prevention and treatment of contrast induced nephropathy after PCI in patients with coronary heart disease and its effect on vascular function. J Hunan Univ Chin Med. (2018) 38(03):335–8. 10.3969/j.issn.1674-070X.2018.03.025

[24] Guo J Yong Y Aa J Cao B Sun R Yu X Compound Danshen dripping pills modulate the perturbed energy metabolism in a rat model of acute myocardial ischemia. Sci Rep-UK. (2016) 6(1):37919. 10.1038/srep37919 PMC5131350 27905409

[25] Bolignano D Donato V Coppolino G Campo S Buemi A Lacquaniti A Neutrophil gelatinase-associated lipocalin (NGAL) as a marker of kidney damage. Am J Kidney Dis. (2008) 52(3):595–605. 10.1053/j.ajkd.2008.01.020 18725016

[26] Gharishvandi F Kazerouni F Ghanei E Rahimipour A Nasiri M. Comparative assessment of neutrophil gelatinase-associated lipocalin (NGAL) and cystatin C as early biomarkers for early detection of renal failure in patients with hypertension. Iran Biomed J. (2015) 19(2):76–81. 10.1128/AAC.47.12.3917-3925.2003 25864811 PMC4412917

[27] Shen SJ Hu ZX Li QH Wang SM Song CJ Wu DD Implications of the changes in serum neutrophil gelatinase-associated lipocalin and cystatin C in patients with chronic kidney disease. Nephrology (Carlton). (2014) 19(3):129–35. 10.1111/nep.12203 24397346

[28] Xiang D Zhang H Bai J Ma J Li M Gao J Clinical application of neutrophil gelatinase-associated lipocalin in the revised chronic kidney disease classification. Int J Clin Exp Pathol. (2014) 7(10):7172–81. PMID: 25400814 PMC4230115

[29] Yan S Zhang X Zheng H Hu D Zhang Y Guan Q Clematichinenoside inhibits VCAM-1 and ICAM-1 expression in TNF-α-treated endothelial cells via NADPH oxidase-dependent IκB kinase/NF-κB pathway. Free Radic Biol Med. (2015) 78:190–201. 10.1016/j.freeradbiomed.2014.11.004 25463279

[30] McMaster WG Kirabo A Madhur MS Harrison DG. Inflammation, immunity, and hypertensive end-organ damage. Circ Res. (2015) 116(6):1022–33. 10.1161/CIRCRESAHA.116.303697 25767287 PMC4535695

[31] Miyoshi A Koyama S Sasagawa-Monden M Kadoya M Konishi K Shoji T JNK and ATF4 as two important platforms for tumor necrosis factor-α-stimulated shedding of receptor for advanced glycation end products. FASEB J. (2019) 33(3):3575–89. 10.1096/fj.201701553RR 30452882

[32] Czarna M Jarmuszkiewicz W. Role of mitochondria in reactive oxygen species generation and removal; relevance to signaling and programmed cell death. Postepy Biochem. (2006) 52(2):145–56. 17078504

[33] Ismail T Kim Y Lee H Lee DS Lee HS. Interplay between mitochondrial peroxiredoxins and ROS in cancer development and progression. Int J Mol Sci. (2019) 20(18):4407. 10.3390/ijms20184407 31500275 PMC6770548

[34] Zorov DB Juhaszova M Sollott SJ. Mitochondrial reactive oxygen species (ROS) and ROS-induced ROS release. Physiol Rev. (2014) 94(3):909–50. 10.1152/physrev.00026.2013 24987008 PMC4101632

[35] Liu K Zhou LY Li DY Cheng WJ Yin WJ Hu C A novel rat model of contrast-induced nephropathy based on dehydration. J Pharmacol Sci. (2019) 141(1):49–55. 10.1016/j.jphs.2019.09.003 31611174

[36] Fang D Wang Y Zhang Z Yang D Gu D He B Calorie restriction protects against contrast-induced nephropathy via SIRT1/GPX4 activation. Oxid Med Cell Longev. (2021) 2021:2999296. 10.1155/2021/2999296 34712381 PMC8548166

[37] Tsamouri MM Rapti M Kouka P Nepka C Tsarouhas K Soumelidis A Histopathological evaluation and redox assessment in blood and kidney tissues in a rabbit contrast-induced nephrotoxicity model. Food Chem Toxicol. (2017) 108(Pt A):186–93. 10.1016/j.fct.2017.07.058 28774741

[38] Mamoulakis C Fragkiadoulaki I Karkala P Georgiadis G Zisis IE Stivaktakis P Contrast-induced nephropathy in an animal model: evaluation of novel biomarkers in blood and tissue samples. Toxicol Rep. (2019) 6:395–400. 10.1016/j.toxrep.2019.04.007 31080747 PMC6506864

[39] Topal I Özdamar MY Catakli T Malkoc İ Hacimuftuoglu A Mamoulakis C Renoprotective effect of taxifolin in paracetamol-induced nephrotoxicity: emerging evidence from an animal model. J Clin Med. (2023) 12(3):876. 10.3390/jcm12030876 36769524 PMC9917797

[40] Iordache AM Docea AO Buga AM Zlatian O Ciurea ME Rogoveanu OC Sildenafil and tadalafil reduce the risk of contrast-induced nephropathy by modulating the oxidant/antioxidant balance in a murine model. Food Chem Toxicol. (2020) 135:111038. 10.1016/j.fct.2019.111038 31825855

[41] Hamasaki S Tei C. Effect of coronary endothelial function on outcomes in patients undergoing percutaneous coronary intervention. J Cardiol. (2011) 57(3):231–8. 10.1016/j.jjcc.2011.02.003 21398093

[42] Lv L Yuan X Jiang L. Effects of compound Danshen dropping pills on adverse cardiovascular events and quality of life after percutaneous coronary intervention in patients with coronary heart disease: a protocol for systematic review and meta-analysis. Medicine (Baltimore). (2022) 101(8):e28994. 10.1097/MD.0000000000028994 35212312 PMC8878783

[43] Zhang Y Ma Y Yang S Liu Y. Effect of compound Danshen dripping pills on contrast nephropathy and prognosis of patients after percutaneous coronary intervention Δ. Chin Pharmacy. (2021) 32(15):1880–4. 10.6039/j.issn.1001-0408.2021.15.15

